# Yolked Oocyte Dynamics Support Agreement between Determinate- and Indeterminate-Method Estimates of Annual Fecundity for a Northeastern United States Population of American Shad

**DOI:** 10.1371/journal.pone.0164203

**Published:** 2016-10-07

**Authors:** Richard S. McBride, Rosalia Ferreri, Emilee K. Towle, Jason M. Boucher, Gualtiero Basilone

**Affiliations:** 1 NOAA Fisheries, Northeast Fisheries Science Center, Woods Hole, Massachusetts, United States of America; 2 Institute for Coastal Marine Environment of National Research Council (IAMC-CNR), Detached Units of Capo Granitola, Campobello di Mazara (TP), Italy; 3 NOAA Fisheries affiliates, under contract with Integrated Statistics, Woods Hole, Massachusetts, United States of America; Shanghai Ocean University, CHINA

## Abstract

Reports of American shad fecundity identify two important themes regarding egg production in fishes. First, geographic variation occurs and is biologically meaningful. Shad annual fecundity decreases with increasing latitude, but predicted lifetime fecundity does not, because of a counter-gradient of survival probability, all of which can explain the adaptive significance of natal homing. Second, the appropriate method of measuring fecundity depends on the pattern of oocyte development. Historically, the relatively simple determinate-fecundity method was used; however, a recent study in a Virginia river indicates that this method may be biased, requiring the more complicated indeterminate method. We address both themes with collections from the 2015 shad spawning run in the Connecticut River, USA. Criteria for using a determinate method were satisfied for this northern population: 1) a size gap evident in the oocyte size frequency distribution, indicating group-synchronous development of yolked oocytes; 2) a decline, early in spawning, in the standing stock of yolked oocytes; and 3) low levels of atresia at the end of spawning. The determinate-method estimate of American shad annual (2015) fecundity (303,000 ± 73,400; mean ± sd) overlapped historic estimates for this and a neighboring river. The indeterminate-method estimate of annual (2015) fecundity (311,500 ± 4,500 sd) was not significantly different from the determinate-method estimate (Student’s t-test, P > 0.05). In contrast, indeterminate-method estimates of annual fecundity for a Virginia population were twice as high as that measured by the determinate method in the past. This can all be explained by fundamentally different patterns of oogenesis (i.e., group synchrony versus asynchrony with respect to yolk development) at different latitudes. American shad, which is distributed within its native range from the Canadian maritimes to Florida, USA (50–30°N), may be particularly well suited to evaluate intra-specific variation in oocyte development, a relatively unexplored life history trait.

## Introduction

Synchrony of oocyte development underlies the reproductive life history of fishes, occurring in three principal patterns. The simplest pattern is complete synchrony: a single cohort of germs cells advances from primordial oogonia to the unfertilized egg during the life time of an individual. This pattern is associated with semelparity, a rare life history pattern among fishes [1].

**Fig 1:**
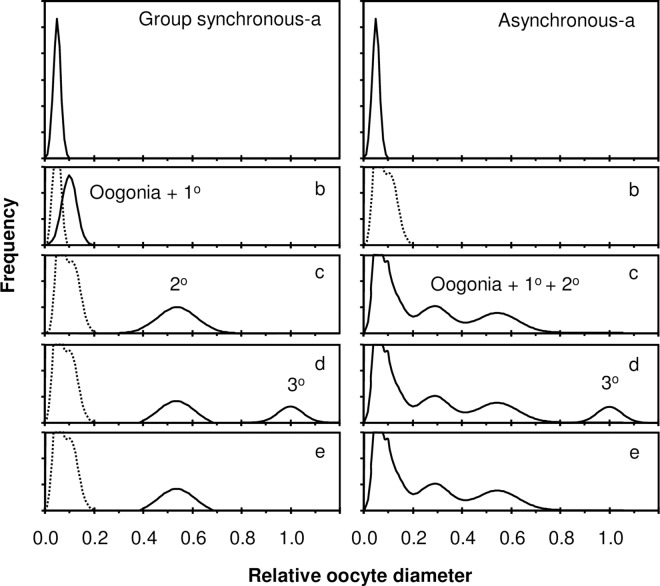
Synchrony of oocyte development with respect to vitellogenesis differs between fish with group-synchronous (left) and asynchronous (right) oocyte development. Time is represented by discrete stanzas from the immature class (a, b), to the mature, pre-spawning class (c), to the spawning class (d, e). Prior to vitellogenesis, the pattern for each type of synchrony is the same among immature females (a,b): germ cells are initially composed of oogonia (a) and then primary oocytes emerge (b; oogonia + 1°, where the truncated dotted mode [right] represents a reservoir of both oogonia and previtellogenic oocytes that persists from year to year in iteroparous fishes). Once vitellogenesis occurs, it can occur in two patterns, each with different consequences. With group synchrony (left), annual fecundity equals the standing crop of vitellogenic (2°) oocytes once the 2° oocytes are readily distinguished (i.e., completely larger) from 1° oocytes but before spawning begins (c). Once spawning begins (d), the standing crop of 2° oocytes diminishes (e) as some cells become tertiary (= mature; 3°) oocytes, either in batches (shown here) or in one uninterrupted wave (i.e., total spawning, not shown). With asynchrony (right), annual fecundity cannot be determined at a single point in time because the standing crop of secondary oocytes is replenished during the spawning periods (i.e., de novo vitellogenesis). Oocyte size is scaled relative to a relative egg size of 1.0.

More common, and the subject of this paper, are group synchrony and asynchrony, characteristic of iteroparous species or populations. In its original conceptualization, Marza [2] focused on the synchrony of yolk provisioning within the oocyte [3]. At the transition from a previtellogenic to vitellogenic (yolked) oocyte stage, oocytes can advance as a single, distinct cohort (group synchronous) or as many, overlapping cohorts (asynchronous) within the spawning period (Fig 1).

The patterns of yolked oocyte synchrony are well established for many species [1, 4]. In the case of American shad, *Alosa sapidissima*, an anadromous herring (Clupeidae) native to the east coast of North America [5], Lehman [6] collected females prior to spawning in the Hudson River and described a pattern of group synchrony. Specifically, Lehman referred to the previtellogenic oocyte stages as primordial “anlagen,” characterizing these as “minute specks of approximately 0.1 millimeter in diameter,” and distinct from the vitellogenic and mature ova, which range in size from 0.4 to 2.1 mm. Implicit in Lehman’s [6] characterization is a gap in oocyte sizes, approximately 100–400 μm. This size gap separates the previtellogenic oocytes from a group of advanced-staged, vitellogenic oocytes that grow, mature, and ovulate within the spawning season. Lehman [6] also reported very low numbers of yolked oocytes found in ‘spawned-out’ females taken at the end of the spawning run.

Distinguishing between group synchrony and asynchrony is a prerequisite for estimating fecundity [7, 8]. Estimating annual fecundity is relatively straightforward for fishes with group-synchronous oocyte development because the standing crop of yolked oocytes equals the annual productivity of that individual fish. Operationally, once the yolked oocytes have become fully recruited (i.e., unambiguously larger than the smaller anlagen), and before ovulation releases any oocytes from the ovigerous lamellae, a maximum annual fecundity can be determined. In the fisheries literature, this is referred to as applying a determinate method to estimate reproductive potential [1, 7, 8].

Alternatively, for fishes with asynchronous oocyte development, the standing crop of yolked oocytes is replenished while spawning is ongoing (i.e., de novo vitellogenesis) [9]. Using a determinate method for such fishes underestimates annual fecundity, at both the individual and population level [10]. Instead, an indeterminate fecundity method is appropriate. An indeterminate method estimates annual fecundity from the individual elements of reproductive potential (i.e., spawning period, spawning interval, batch fecundity).

Gordo et al. ([11] and references therein) outlined three criteria for whether a determinate method estimate of annual fecundity is unbiased: 1) observing the oocyte size frequency distribution, specifically for a size gap that clarifies whether yolked oocyte development is group synchronous or not; this is a practical necessity so that the standing crop of yolked oocytes can be accurately counted; 2) testing if the standing crop of yolked oocytes declines early in spawning; the determinate method assumes that the standing crop of oocytes declines throughout the spawning period in the absence of new recruitment of yolked oocytes, but if not, then annual fecundity would be underestimated; and 3) evaluating the levels of atresia at the end of the spawning period; the determinate method assumes that there are no appreciable atretic but unovulated advanced oocytes remaining in the ovary at the end of the run, but if so, then annual fecundity would be overestimated. Lehman [6] appears to satisfy the first and third criteria; however, Lehman's description of group synchronous oocyte development in American shad, and the application of the determinate fecundity method to American shad populations (e.g., [12]), has not been scrutinized for a half a century [13, 14].

Recently, Hyle et al. [14] demonstrated that some but not all female American shad from the York River, Virginia, population exhibited a group synchronous pattern. In addition, Ganias et al. [15] documented that yolked oocyte development of a Connecticut population of alewife, *Alosa pseudoharengus*, occurs asynchronously and large numbers of yolked oocytes remained in ovaries of females leaving the spawning grounds. Synchrony of oocyte development varies considerably among Clupeiformes [1], and these two studies [14, 15] suggest that both intra- and inter-specific variation occurs within the genus *Alosa*. American shad is also a particularly interesting species to examine, because this potential variation in oocyte synchrony may co-vary with variation in other life history traits, including maximum age, probability of repeat spawners, and annual fecundity, all of which vary with latitude [13].

Our investigation began in 2014, when we collected American shad from two northern rivers–the Connecticut and Merrimack rivers–and examined oocyte size distributions (S1 Material). The results were indicative of a group synchronous pattern of oocyte development in both systems, but sampling occurred only on three dates at one location in each river, such that an additional, independent sampling effort was deemed necessary. In 2015, sampling was designed to collect female American shad throughout the Connecticut River spawning run: 1) in the lower river at the earliest part of the run (late April); 2) at three other sites along the river; and 3) among the down-running, post-spawning females at the end of the run (late June) at two sites already sampled along river.

Using these collections from the 2015 spawning run, we set out to evaluate all three assumptions of the determinate fecundity method, and once these criteria had been satisfied, to re-estimate annual fecundity for the Connecticut River population of American shad. The determinate method is well established and has received useful technological updates. Historically, oocytes were arduously counted and measured by hand with an ocular micrometer (e.g., [16]). Today, this work is more productive, aided by automation and image analysis [17], so that estimates of fecundity and determination of the synchrony of oocyte development should be routine in reproductive studies. Relevant to our objectives, such methodology estimates potential annual fecundity (PAF) by counting the standing crop of an advanced cohort of oocytes present at the beginning of the spawning period, assuming that all oocytes counted will ovulate by the end of the spawning run.

The indeterminate method is also applied here. It does not assume that all yolked oocytes are recruited at the beginning of the spawning period nor that all yolked oocytes are ovulated by the end of the spawning period. Instead, it estimates annual fecundity as a product of the basic components of reproductive effort, namely the number of spawning events and the number of eggs produced per spawning event. It has more data requirements, because more parameters need to be estimated, so it may not be as practical as the determinate method. Still, it provides an independent estimate of annual fecundity, although to our knowledge, no estimates of annual fecundity by both methods have been reported in a single study (see also [18]).

Estimating annual fecundity by both methods has three purposes here: 1) to validate that group synchrony is the dominant mode of yolk provisioning in the Connecticut River population of American shad; if it is not, then the independent fecundity estimates will not agree; 2) that the relatively cheaper, easier determinate fecundity method is sufficiently accurate to use routinely, at least for this population; and 3) to determine how much, if by any, these recent estimates of annual fecundity differ from historic reports. And because this pattern of yolk provisioning is different than a recent report in a Virginia river [14], we comment on the likelihood and context that American shad’s pattern of oogenesis varies along a latitudinal gradient.

## Materials and Methods

### Field methods

Female American shad were collected in the Connecticut River during the 2015 spawning run. Briefly, sampling began using gill nets in the lower river, near Old Lyme Connecticut ([CT] 41.3°N, 72.3°W, river kilometer [rkm] 5), on April 30, 2015. Pre-spawned females were also purchased from a commercial gill net fisherman who sampled at night (May 5–6) from rkm 24 to 34 (near Chester, CT). Up-running spawning migrants were collected between mid-May and mid-June from Massachusetts and southern Vermont portions of the river; these were dipped from fish traps, each part of fishway systems located at Hadley Falls (rkm 135), Cabot (rkm 191), and Vernon (rkm 228) Power Stations (see [19] for map). Down-running post-spawning migrants were collected from mid-June at Hadley Falls Power Station and mid- to late June at Cabot Power Station. Fish were collected during the morning (0900–1230), kept cool, and processed within six hours to ensure the quality of the reproductive tissue. Sampling conformed to the scientific collection permits issued by the states of Connecticut, Massachusetts, and Vermont, and training and permissions required by each power station.

### Initial fish processing

Fork length (*L*_F_; ±1 mm), total body mass (*M*_T_; ±0.1 g), and ovary mass (*M*_O_; ±0.001g) were measured, and spawning activity was evaluated using the reproductive investment equation of the gonad-somatic index (*I*_G_) = 100 *M*_O_ (*M*_T_ − *M*_O_)^−1^. Macroscopic classification of the gonad followed Olney et al. [20]: (A) developing (filled with yolked eggs, no evidence of spawning [was called maturing in the original paper]), (B) hydrated (hydrated oocytes scattered throughout yolked oocytes), (C) running ripe (a batch of hydrated oocytes present in the ovary lumen), (D) partially spent (similar to developing, but a smaller gonad, as a result of several spawning events), (E) spent (a small-moderate sized, post-spawning ovary that was flaccid and may have had some advanced remnant oocytes, either yolked or hydrated), and (F) resting (a small ovary with no advanced oocytes). The sagittal otoliths were removed, and fish age, in years, was estimated from counts of annual band pairs from whole otoliths [21, 22].

### Gonad histology

**Fig 2:**
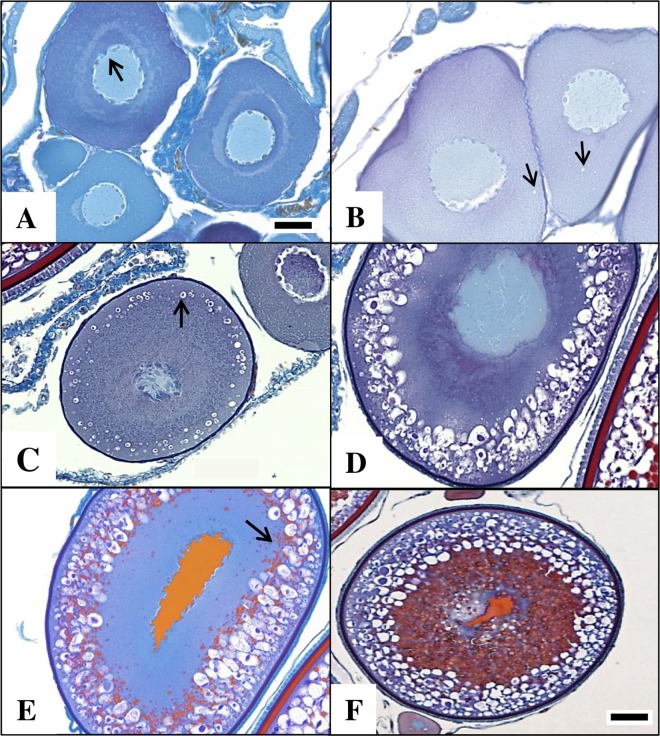
A sequence of oocyte stages depicting the transition from primary to secondary oocytes. **A.** Perinucleolar (PE)–thin chorion, no cytoplasmic inclusions; nucleoli visible around periphery of nucleus (arrow). **B.** Early cortical alveolar (C1)–thin chorion; tiny clear inclusions appear around cytoplasm (arrow). **C, D.** Late cortical alveolar (C2)–chorion thickening; dark inclusions appear within the white inclusions of the cytoplasm (arrow). Image D is more advanced; the cortical alveoli grow larger and proximally. **E.** Early vitellogenesis (V1)–first sign of yolk (red) inclusions appear (arrow). As the cell grows, yolk continues to fill the cytoplasm. **F.** Cell is still staged as V1 as long as yolk has not expanded to the distal edge of the cytoplasm. Black scale bar: images A–E bar = 50 **μ**m; image F bar = 250 **μ**m.

Since histological analysis allows greater accuracy than macroscopic methods to estimate ovarian spawning phases [9, 23], germ cell development was confirmed by examining ovary tissue fixed in formalin and prepared according to standard paraffin embedding techniques. A Schiffs-Mallory trichrome stain was used [24, 25] and histology slides were viewed on a large monitor using a microscope and digital camera system. The earliest oocyte stage recorded was perinucleolar (PE) (Fig 2A). Two subsequent secondary growth oocytes stages distinguished between a more developed germ cell with small, clear cortical alveoli scattered in the cytoplasm (C1) and a later stage where the cortical alveoli appeared more numerous, larger and with dark inclusions within the cortical alveoli (C2) (Fig 2B, 2C and 2D). Two vitellogenic stages were observed, an early stage where the yolk partially filled the cytoplasm (V1) (Fig 2E and 2F) and a later stage where the yolk completely filled the cytoplasm (V2), except along the distal rim where the cortical alveoli became concentrated. Three stages of nucleus migration (NM) were observed–an early (NM1), middle (NM2), and late stage (NM3)–as characterized by Hyle et al. [14], along with a hydrated stage both within (H) and outside (O) the follicle, the latter occurring once ovulated. These shortened labels (i.e., PE, C1, C2, etc.) are used when referring to oocyte stage as viewed by gonad histology.

The size distribution of each oocyte stage was determined by measuring the diameters of individual cells with a calibrated scale bar, using projected images of histology slides on a computer monitor. To minimize measurement bias, a cell was only measured if the section included its nucleus; in the advanced stages of maturation, when the nucleus could not be observed, the measurements are biased but they are included here for relative comparison. Some additional bias occurs at all stages as a result of dehydration of the germs cells, a part of standard histology procedures; this would be most pronounced in the hydrated cells, whether in the follicle or already ovulated. These nominal measures of oocyte diameters, by stage (histology), are included to compare to diameters measured from whole oocytes (see next section and Table 1).

**Table 1:** Characteristics of whole oocytes, by phase of development, and corresponding histology stages. Appearance is as under transmitted light. Sizes are approximate (100 μm). See text and Fig 2 for description of histology stages.

Whole mount oocyte phase	Size, appearance	Histology stage
Primary growth	Small (< 300 μm), transparent	PE, C1
Transitional growth	Medium (300–500 μm), translucent (gray)	C1, C2, V1
Secondary growth	Large (500–1500 μm), opaque (black)	V1, V2, NM1
Oocyte maturation	Largest (1200–2000 μm),translucent–transparent	NM2, NM3, H, O

Post-ovulatory follicles (POFs) were also identified following the criteria outlined by Hyle et al. [14]; herein, we only report the presence or absence of POFs, as an indication of whether spawning had started in an individual fish. Oocytes were also evaluated for atresia, particularly for beta-atresia of yolked oocytes (depicted in [26]), which if widespread at the end of the spawning season, would violate one of the assumptions of determinate fecundity.

### Identifying oocyte synchrony

Whole oocyte diameters were measured from randomly-selected upstream-migrating, developing (Class A) females collected at all major sampling points, as well as from downstream-migrating, spent or resting females (Classes E, F) collected at the end of the spawning run (Table 2). A subsample of fixed ovarian tissue was teased apart and the germ cells were evenly distributed into small, water-filled wells. Enough tissue was taken to capture at least 100 vitellogenic germ cells in a digital image using a Leica MZ6 scope and DFC295 camera. The diameters of these cells were automatically measured using a macro installed into Image J (ver 1.48v, National Institute of Health) and the Object J plugin (ver 1.03p, University of Amsterdam). A total of four whole oocyte phases were identified (Table 1), and measurements and counts of the non-vitellogenic phases were measured manually (Fig C in S1 Material). Oocytes smaller than 100 μm were not well represented in the graphic depictions because they were difficult to separate from each other and because the macro does not efficiently identify them. We do not consider this a problem, because we are focused on the dynamics of oocytes in the 200–500 μ range. The whole oocyte diameters, by phase, were plotted as histograms for selected individual females.

**Table 2:** Characteristics of individual females selected for measurements of oocyte diameters and the corresponding figure they appear in. Data include: Sampling location along the Connecticut River, sampling date (2015), fish length (fork length [*L*_F_], mm), fish age (years, otolith method, one age not available), gonad-somatic index (*I*_G_), and histology details (i.e., most advanced oocyte stage [MAOS, see text for definitions] and whether post-ovulatory follicles [POFs] were present [+] or absent [-]).

A) 2015 Upstream Migrants							
**Location**	**Date**	***L***_**F**_ **(mm)**	**Age (yr)**	***I***_**G**_	**MAOS**	**POFs**	**Panel**
Lower River	Apr 30	426		21.5	NM2	-	A
Lower River	Apr 30	467	5	16.4	NM1	-	B
Lower River	May 05	528	7	8.5	V2	-	C
Vernon	May 20	449	5	20.0	NM2	+	D
Cabot	Jun 10	401	3	27.1	NM2	-	E
Hadley Falls	May 19	503	6	23.7	NM1	-	F
B) 2015 Downstream Migrants							
**Maturity Class**	**Date**	***L***_**F**_ **(mm)**	**Age**	***I***_**G**_	**MAOS**	**POFs**	**Panel**
Spent (E)	Jun 18	408	5	2.6	NM1	+	A
Spent (E)	Jun 18	426	5	3.2	NM1	+	B
Spent (E)	Jun 30	458	7	4.5	NM1	+	C
Resting (F)	Jun 18	423	5	1.9	V2	+	D
Resting (F)	Jun 30	422	4	1.7	V1	+	E
Resting (F)	Jun 30	417	5	1.5	PE	+	F

### Determinate fecundity estimation

A determinate-method PAF was estimated by counting the standing crop of the advanced cohort of oocytes. Based on the examination of whole oocytes (see Results), this advanced cohort was defined as transitional and secondary oocytes (C1, C2, V1, V2, NM1; Table 1). These fish were classified macroscopically as developing (Class A), and presence of these oocyte stages, and not more advanced stages, was confirmed by examining gonad histology. Samples without POFs were categorized as pre-spawning and those with POFs were categorized as spawning females.

Determinate PAF was estimated for 45 pre-spawning females: 25 collected in the lower river by gillnet (4/30–5/6) and 20 collected early in the run at Hadley Falls (5/12). Annual fecundity was also estimated from 21 spawning females: 11 collected early in the run at Hadley Falls and Cabot Power Stations (5/12–5/13), and 10 collected at Hadley Falls and Vernon Power Stations one week later (5/19–5/20). Estimates of PAF from pre-spawning and spawning females were compared with analysis of covariance (ANCOVA) to confirm that the standing crop of oocytes declined with spawning, which would be predicted if fecundity was determinate at the beginning of the spawning run. All fish ranged in size from 396 to 528 mm *L*_F_ (1023.8g–2011.3 g *M*_T_), with *I*_G_ between 8.5 and 22.6.

Determinate PAF was estimated gravimetrically (PAF = [*C*_S_ / *M*_S_] * *M*_O_), excising one subsample of tissue (0.1975–0.5564 g [*M*_S_]), which produced cell counts between 138 and 532 (*C*_S_). A macro (ImageJ software: v1.48v, ObjectJ software: 1.03p) automatically measured a majority of the oocytes, which was followed by inspection to ensure accuracy (e.g., when cells were touching).

Initially, the counts of transitional growth phase cells were kept separate from both the other primary growth phase cells and the secondary growth phase cells. After analyzing the oocyte length frequencies, it was decided that the transitional growth phase observed at the beginning of the spawning run recruit fully and are spawned in the current year. Their numbers are both included in and omitted from the determinate PAF estimate, to show that even when they are present, they are typically a small percentage of annual fecundity. The percentage of transitional growth oocytes, among transitional and secondary oocytes combined, is referred to later as “gap uncertainty,” where higher values indicate higher uncertainty in group synchrony of yolk oocyte development and potential for bias in the annual fecundity estimate when using a determinate method.

### Indeterminate fecundity estimation

We simulated indeterminate PAF following the method of Hyle et al. [14]. Using the software R (version 3.0.3, R Core Team, 2014), PAF was calculated according to the equation: $PAF=\frac{RT}{SI}\times BF$, where RT is the residence time, SI is the spawning interval, and BF is the batch fecundity. Residence time (RT; mean = 30.0, sd = 2.6) on the spawning grounds in the Connecticut River was based on hatchery observations and preliminary tagging analyses (T. Castro-Santos, personal communication). SI estimates were calculated for 12 sampling dates, as the reciprocal of the fraction of females spawning daily; spawners were classified in relation to days to or since the most recent spawning date based on gonad histology [14]. Batch fecundity (BF) values were based on the counts of hydrated oocytes in 15 gravid females. An improvement to the method [14] was made in the estimation of parameters for the SI and RT probability density functions using a maximum likelihood estimation procedure. Comparisons of PAF estimates by the determinate versus the indeterminate method were made with a Student’s t-test.

### Historical (determinate) fecundity estimates

Lehman’s [6] “green” fish are the equivalent to our “pre-spawning” females. In the field, Lehman only sampled fish in the lower river, prior to their arrival on the spawning grounds, whereas we collected fish in the lower river, and of those collected at the power stations, we separated pre-spawning from spawning fish using the absence and presence of POFs, respectively. Lehman did not have the benefit of image analysis to automate the process, but this gave us only the advantage to speed up sample processing, and should not have contributed to quantitative differences. Lehman’s calculation of PAF (i.e., PAF = [*C*_S_ / *M*_S_] * *M*_O_) is the same as ours. We also comment on an estimate from a small sample reported by Davis [12], using the same methods. Together [6, 12], these values are a relevant historic benchmark of PAF from the Hudson River, a neighbor of the Connecticut River.

Historical data for the Connecticut River were also available, specifically William Leggett’s dissertation and the subsequent peer-review summary [27, 28], where fecundity was calculated by grading the oocytes into different size groups, using sieves, and expanding the estimate gravimetrically. Leggett and his collaborators also collected only pre-spawning females and counted oocytes as small as 250 μm; therefore their estimates should be comparable to Lehman [6], Davis [12], as well as the new (2015) data for the Connecticut River.

One bias that might be expected among historical material is the greater presence of larger and older repeat spawners that could have higher annual fecundity. In contrast, repeat spawning was low in 2015 (< 2%, based on scale morphology), so these estimates were for virgin spawners. Although this does not seem to influence our conclusions, we report, where possible, fecundity in relation to size or age to account for this potential bias.

## Results

### Dynamics of oocyte development

**Fig 3:**
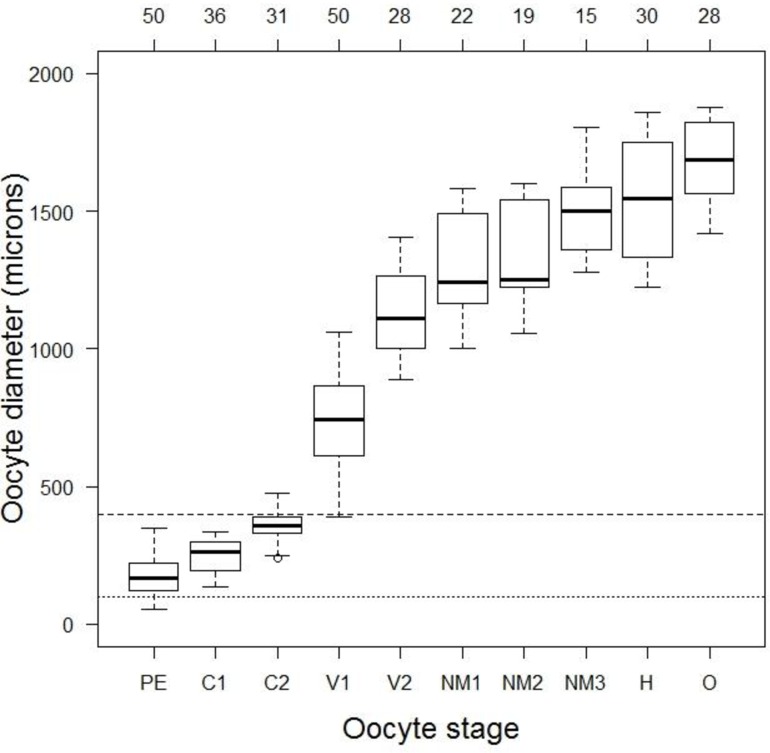
Box-whisker plots of oocyte sizes, as measured from histology preparations. For each of ten oocyte stages (see text and Table 1 for full stage descriptions), the data are represented by a thick horizontal line (median), a box (25–75^th^ percentile), whiskers (range), and any outliers (then the whiskers are roughly the 95% confidence limits). The lower (dotted) horizontal line corresponds to the size (100 μm) of the primary growth (‘anlagen’) oocytes, and the upper (dashed) line corresponds to the lower size (400 μm) of vitellogenic oocytes, as reported by Lehman [6] and assumed by others (e.g., [12]). Values along the top axis are numbers of cells measured by stage. See Fig 2 for images of the first four stages.

**Fig 4:**
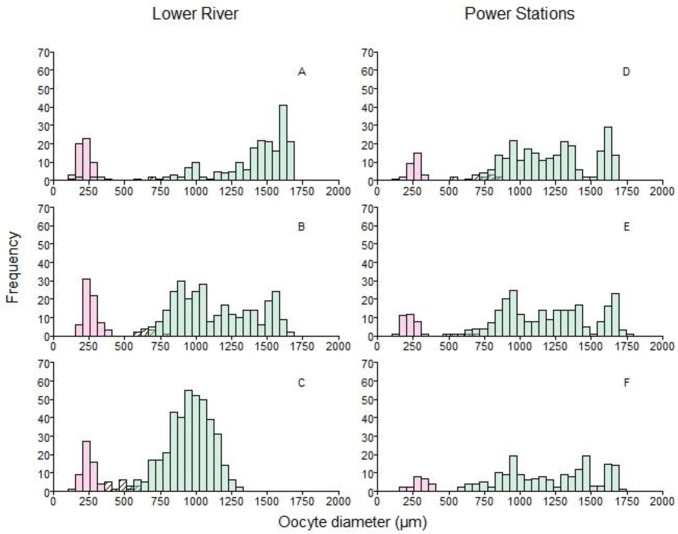
Histograms of oocyte diameters from six upstream migrating, female American shad collected from the Connecticut River in 2015 (one fish per panel). All females were developing (Class A). Fish in panels A-C were collected by gill net in the lower river, whereas fish in panels D-F were collected at three different power stations: Vernon (D), Cabot (E), and Hadley Falls (F). See Table 2A for more details about each fish. Colors are transparent and overlaid (not stacked), corresponding to small, transparent oocytes (red; primary growth phase), medium, translucent oocytes (yellow; transitional growth phase), and larger, opaque oocytes (green; secondary growth phase). Angled hatching is overlaid on the yellow bars to distinguish them more. See Table 1 for additional details about phases of development.

**Fig 5:**
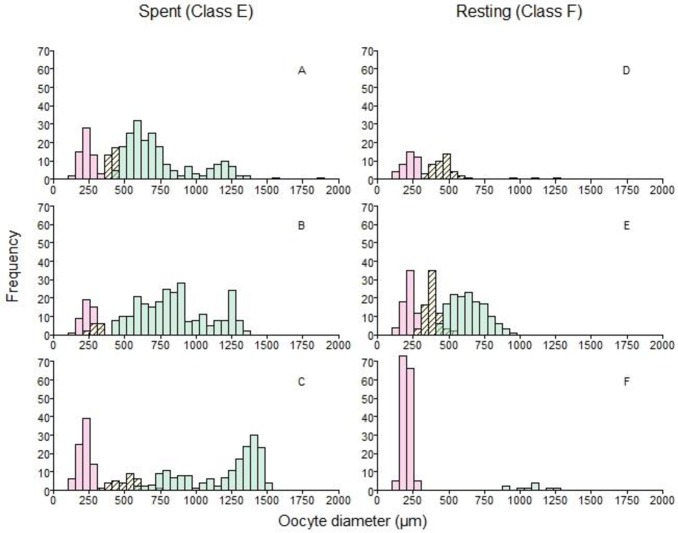
Histograms of oocyte diameters from six downstream migrating, female American shad collected from the Connecticut River in 2015. Fish on the left (panels A-C) were spent, whereas fish on the right were resting (D-F). At least one sample of both maturity classes shown were collected on both June 18 at Hadley Falls Power Station and June 30 at Cabot Power Station. See Table 2B for more details about each fish. Colors and hatching identifying each growth phase are the same as in Fig 4.

Microscopically, all expected oocyte stages were observed–previtellogenic, vitellogenic, and mature (Figs 2 and 3)–including cells with cortical alveoli but no evidence of yolk inclusions. Cells with cortical alveoli covered the size range of at least 300–400 μm, which had not been mentioned by Lehman [6]. Nonetheless, when present, these stages (C1, C2) that define the transitional phase occurred in low numbers (i.e., low gap uncertainty [0–8%]; see next section for details), (Figs 4 and 5).

The process of group synchrony was more apparent in a dynamic context, when histograms measured at different points of the spawning run were compared. In the lower river, early in the run when females had not started spawning (i.e., no POFs), there was either a complete size gap or a reduced number of transitional phase oocytes (Fig 4). By the time females had reached the sampling sites further upstream (i.e., power stations; Fig 4), where spawning was first detected, the size gap was more obvious. This indicated that transitional growth oocytes, comprised mostly of the cortical alveolar staged oocytes (C1, C2), had advanced to the secondary growth phase, which in turn, indicates that de novo recruitment of yolked oocytes has stopped by the time females reached the spawning grounds.

In some spent (Class E) females, cortical alveolar staged oocytes were present (Fig 5A–5C) but these cells appeared to be part of a fresh cohort developing for next year. In some resting (Class F) females, cortical alveolar and fully vitellogenic oocytes were present as well, but the fate of each stage appears to be very different: the cortical alveolar stages (C1, C2) are recrudescing for next year’s spawning stock of oocytes (Fig 5D), whereas the fully vitellogenic (V2) stages were few in number and atretic (Fig 5F). In one female, early vitellogenic (V1) oocytes were the most advanced stage, but these were not atretic and appeared to be the advancing edge of the germ cell cohort developing for next year’s spawning run (Fig 5E).

### Determinate fecundity estimates

**Fig 6:**
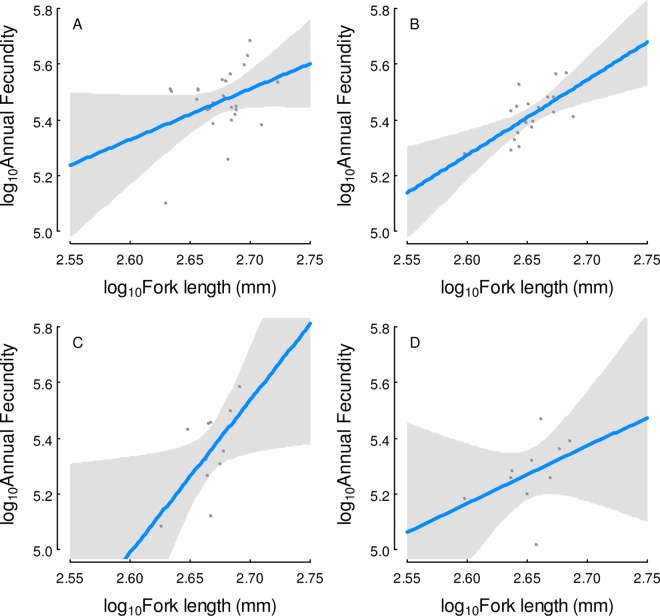
Estimation of potential annual fecundity, as measured by a determinate fecundity method, relative to fish size (log-log transformation). The size-specific estimate decreases once a fish shows evidence of spawning (see text). The four sampling events and associated correlation coefficients are: A) pre-spawning fish in the lower river (April 30–May 6, *r* = 0.36, *n* = 25, *P* = 0.08), B) pre-spawning fish collected at Hadley Falls Power Station (May 12, *r* = 0.65, *n* = 20, *P* = 0.002), C) spawning fish collected at Hadley Falls and Cabot Power Stations (May 12–13, *r* = 0.63, *n* = 11, *P* = 0.04), and D) spawning fish collected at Hadley Falls and Vernon Power Stations (May 19–20, *r* = 0.41, *n* = 10, *P* = 0.2). Individual fish are plotted as dots, the predictive regression is a blue line, and the 95% confidence limits are enclosed in solid gray.

Mean PAF was highest among pre-spawning females in the lower river (302,800 ± 73,400 [mean ± sd]), where it varied nearly 4-fold (126,400–483,000 [range]) (Fig 6A). It was also high among pre-spawning females collected on 5/12 at Hadley Falls (265,900 ± 52,500; 189,700–370,800) (Fig 6B). Gap uncertainty was relatively low. If the transitional phase oocytes were not counted, then the PAF estimates of pre-spawning females would have been, on average, 4% lower (range: 0–8%) in the lower river and 2% lower (0–8%) at Hadley Falls.

Reduced PAF estimates were evident when applying the determinate method to spawning fish. PAF in spawning fish was lower, 237,600 ± 78,600 (121,700–383,400) at Hadley Falls on 5/12 (Fig 6C), and lowest (194,600 + 53,100; 104,100–293,300 one week later (Fig 6D). Gap uncertainty was low in spawning females as well: transitional phase oocytes were fewer, about 2% (0–7%) of the advanced cohort.

This decline in PAF early in the spawning run was statistically significant. PAF was significantly lower in spawning females, compared to pre-spawning females, based on ANCOVA of log-transformed data. First, there were no significant interaction terms between fecundity, as the response variable, and female size and the four sampled groups (date, location combinations, above), as predictor variables. Thereafter, the model was reduced to main effects, which had significant effects of fish size (*P* < 0.001), and sample group (*P* < 0.01). Contrasts among parameter estimates showed no significant differences in PAF between the two pre-spawning groups, but spawning fish had significantly lower PAF than the pre-spawning fish (*P* < 0.05 on 5/12–13, and *P* < 0.01 on 5/19–20).

### Indeterminate fecundity estimates

**Fig 7:**
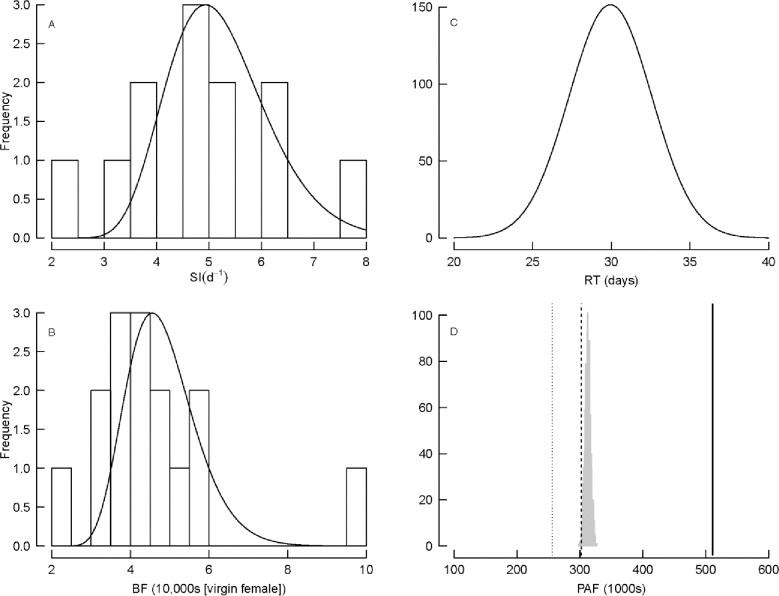
Estimates of reproductive characteristics for female American Shad: (A) spawning interval (SI), (B) batch fecundity (BF), (C) residence time (RT) as a proxy for spawning period, and (D) potential annual fecundity (PAF). Data are plotted as open bars together with simulated distributions (solid curves) in panels (A) and (B). RT is simulated as a normal distribution based on hatchery and tagging observations (panel C). Virgin spawner PAF estimates are plotted in panel (D) as vertical lines for the determinate method for the Connecticut River population in 2015 (303,000; dashed line), the determinate method by Leggett [27] for the Connecticut River (263,000; dotted line), and the indeterminate method by Hyle et al. [14] for the York River (511,000; solid line), together with 1,000 bootstrapped mean estimates of PAF (gray bars) from the simulations of PAF = RT/SI × BF (i.e., the indeterminate method for Connecticut River fish in 2015).

A normal probability density function of RT was simulated from the observed estimates for American shad in the Connecticut River (mean = 30.0, sd = 2.6), producing a range from 23 to 38 days (Fig 7C). Not all fish could be used to calculate SI: fish in the lower river had not begun to spawn, so such fish produced unrealistically high SI estimates (mean 13.99 days ± 6.16 sd); fish moving downriver had typically completed their spawning, and as such they were in a spent or resting condition (Classes E, F). To avoid biasing our simulations, SI estimates were limited to only fish captured moving upriver beginning at Hadley Falls (Table 3). Mean SI was 4.83 days (± 1.46 sd), with a range of 2.44 to 7.69 days (Fig 7A). These values produced a wide range of spawning frequencies during the season, from 2.8 to 18.2 with a mean of 6.7 batches (± 2.1 sd). Mean BF was 45,950 (± 16,670 sd), with a range from 24,140 to 97,200 (Fig 7B). From the simulated population, mean PAF estimated by the indeterminate method using the data from the 2015 spawning migration was 311,500 + 4,500 sd, ranging from 295,600 to 323,300 (Fig 7D).

**Table 3:** Spawning fraction (SF) by sample day, and spawning interval (SI; days), calculated as the inverse of SF, for Connecticut River American shad in 2015. SF was initially calculated as the proportion of fish spawning per day from different histological markers, each adjusted to spawning: the next day (day -1; most advanced oocyte stage [MAOS] = NM1), that day (Day 0; MAOS = NM2, NM3, H, or POF0 [fresh postovulatory follicle]), yesterday (Day +1; POF1 [12–24 hour old POF]), or the previous day (Day +2; POF2 [24–24 hour old POF]). Final estimate of SF was a mean of these separate values. See text and Table 1 for additional explanation of histology codes. Sampling sites are Hadley Falls (H), Cabot (C), and Vernon (V). *N* = number of females sampled for histology by date.

			Proportions by sampling day relative to spawning (Day 0)				Mean	
Dates	Site	*N*	Day -1	Day 0	Day +1	Day+2	SF	SI
May 12	H	28	0.61	0.11	0.00	0.18	0.22	4.54
May 13	C	30	0.57	0.20	0.13	0.37	0.32	3.13
May 19	H	29	0.41	0.38	0.00	0.03	0.21	4.76
May 20	V	32	0.50	0.34	0.03	0.75	0.41	2.44
May 26	H	33	0.21	0.36	0.03	0.03	0.16	6.25
May 27	C	32	0.28	0.34	0.00	0.22	0.21	4.76
Jun 2	H	27	0.44	0.19	0.04	0.07	0.19	5.26
Jun 3	V	5	0.60	0.20	0.00	0.20	0.25	4.00
Jun 9	H	28	0.46	0.11	0.00	0.07	0.16	6.25
Jun 10	C	16	0.19	0.44	0.00	0.13	0.19	5.26
Jun 16	H	32	0.16	0.34	0.00	0.03	0.13	7.69
Jun 17	V	8	0.75	0.38	0.00	0.00	0.28	3.57

### Method comparison

The determinate method makes a number of assumptions that equate the standing crop of oocytes to annual fecundity, whereas the indeterminate method does not. If these assumptions are correct, then there should be no significant difference between PAF estimates calculated by the determinate and indeterminate fecundity methods. Mean PAF with the indeterminate fecundity method was <3% higher than with the determinate fecundity method, and not significantly different (Student’s t-test, *P* < 0.05).

### Historical comparisons

**Fig 8:**
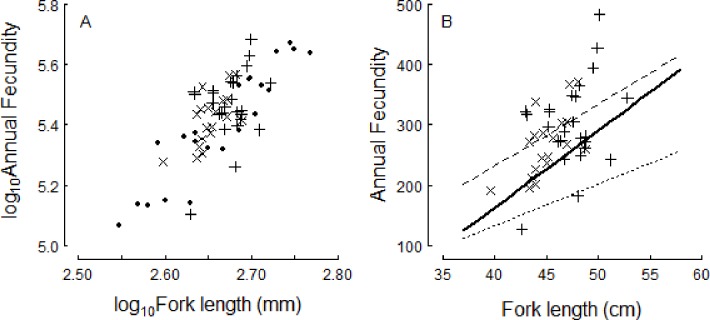
Fecundity-size relationships overlapped between recent and historic collections. Values here are for pre-spawning females in the Connecticut River (2015)–collected in the lower river (plus [+] symbol) or the Hadley Falls Power Station (cross [×])–compared to two historic data sets: A) pre-spawning females sampled from the Hudson River 1951 (dots [.], data from [6]); B) and predictive, linear model parameters reported for three rivers as sampled in the 1960s: York River, Virginia (dashed, thin line), Connecticut River (solid, thick line), and St. John River, Canada (dotted, thin line) (parameters from [27]; table 30). In (B), raw data were not included in Leggett [27], fork length was measured in cm, and fecundity was not log-transformed (compare to Fig 7A).

In relation to historic estimates of fecundity-size relationships among pre-spawning females, recent values from the Connecticut River (2015) overlapped the values reported by Lehman for pre-spawning females collected in 1951 in the Hudson River [6] (Fig 8A). Spawning fish in 2015 were mostly age-5; in comparison, Davis’s [12] independent estimates of annual fecundity of age-5 American shad from the Hudson River (249,000; *n* = 3) were not significantly different than those estimated by Lehman (220,000; *n* = 5) (Welch two-sample t-test, *P* = 0.16). Within the Connecticut River itself, no obvious differences in these relationships were apparent when comparing the recent (2015) data to historic (1965–1973) data (Fig 8B).

## Discussion

In a single study, we investigated the morphological basis of how yolk provisioning proceeds in Connecticut River American shad, we showed that the cost-effective, determinate method to estimate PAF is reliable for this population, and we explored the limited data available regarding the potential for population-specific PAF to vary between decades. These topics are discussed further, below, as well as the emerging understanding that the pattern of American shad oogenesis itself may vary with latitude and what this means for the established narrative that life history variation in this species is adaptive.

Yolked oocyte development is group synchronous in Connecticut River American shad. This was demonstrated by a size gap between primary and secondary oocytes, although the gap was not particularly large early in the spawning run, among pre-spawning females. As a comparison, prior to spawning by Atlantic herring, *Clupea harengus*, the size gap can grow to over 1000 μm ([29]; see also S1 Material). However, Atlantic herring is a total spawner, advancing the entire cohort of vitellogenic oocytes to maturation and through an uninterrupted period of ovulation, whereas American shad is a batch spawner, such that vitellogenic oocytes mature and ovulate in a series of discrete events during an extended spawning run. American shad produce a large egg, and as a batch spawner it need not advance the entire cohort of yolked oocytes at once, so the wide range of its yolked oocyte sizes should not be unexpected.

A complete size gap was not evident in all females examined, which required close inspection. Oocytes with cortical alveoli (stages C1, C2), roughly 300–500 μm in diameter, were present in some females as they enter the river. This was observed in preliminary collections made in 2014 at Hadley Falls (S1 Material) and in 2015; however, this was not expected according to Lehman’s [6] characterization of oocyte distributions in a neighboring river. We regard these differing observations as the result of the lack of resolution of Lehman’s method, whereas our use of digital imaging and image analysis software allowed us to recognize and quantify the low levels of transitional oocytes when they did occur (i.e., gap uncertainty).

Overall, yolked oocyte development was quite dynamic throughout the spawning run. The C1 and C2 germ cells developed into yolked oocytes once spawning commenced and were not replaced immediately; however, they re-appeared at the end of the spawning run, among spent and resting fish (Classes E, F), showing that recrudescence of the gonad for the following year begins in some females before they leave the river during the current spawning year. More detailed characterization of oocyte growth dynamics among cells < 500 μm would require specialized techniques (e.g., [9, 10, 30]). Although such detailed research would be useful to understand recrudescence of the standing stock of yolked oocytes, as well as to independently confirm that de novo vitellogenesis is not occurring, this additional effort would unlikely alter our conclusion that oocyte development in the population is group synchronous, and that a determinate fecundity method can be used routinely for accurately estimating annual fecundity for this population.

Brown-Peterson et al. [31] consider previtellogenic oocytes with cortical alveoli as secondary growth oocytes. We agree, at least relative to the beginning of the spawning run, that these cells will spawn in the current period and should be counted as part of a determinate fecundity method to estimate Connecticut River American shad PAF. Among pre-spawning individuals, these cells averaged only 2–3% (i.e., gap uncertainty) of the secondary oocytes counted using the determinate fecundity method. Not counting them would produce a relatively minor but biased underestimate of PAF. However, according to our interpretation of this population’s oocyte dynamics, the cells with cortical alveoli (i.e., transitional growth phase) present in partially-spent, spent, and resting females are developing for next year’s spawning.

Group synchrony of yolked oocytes was not reported for American shad from a Virginia population [14], for the twaite shad, *Alosa fallax fallax* [26], and for alewife, *Alosa pseudoharengus* [15]. This suggests that the synchrony of oocyte development may be a dynamic trait within the genus *Alosa*. Still, this trait has been poorly examined, particularly in early reports that did not adequately measure the smaller oocytes (e.g., [7, 13, 20]). Synchrony of oocyte development remains relatively unexplored and is worth investigating further because this dynamic life history trait underlies oogenesis itself and it drives the rate of batch spawning frequency and the resulting numbers of eggs produced [1].

Group synchrony is only one expectation of determinate fecundity. Examination of pre-spawning and spawning females collected early in the run demonstrated that fecundity estimates declined immediately once fish started spawning, which is predicted for determinate fecundity. Third, examination of resting (Class F) females late in the run demonstrated the presence of few atretic, yolked oocytes, which was also reported by Lehman [6]. The indeterminate method, which does not have these requirements, is by default more costly because more parameters need to be estimated to calculate annual fecundity. We not only conclude that a determinate fecundity method is valid, but that it is possible to estimate fecundity from fish collected on the spawning grounds, at least when using gonad histology to separate pre-spawning from spawning females, based on the absence or presence of POFs. Doing this allowed us to estimate PAF of fish at the Hadley Falls Power Station. Previously (e.g., [12]), investigators relied on collecting females early in the season, or below spawning areas, to avoid collecting fish that have could have ovulated one or more batches of eggs.

**Fig 9:**
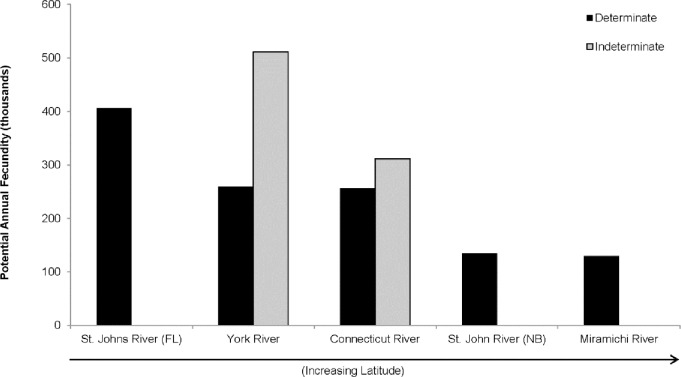
The decreasing trend in potential annual fecundity with increasing latitude, as observed previously with the determinate fecundity method, persists for the two rivers analyzed with the indeterminate fecundity method. This figure compares determinate fecundity estimates from Leggett and Carscadden [28], indeterminate fecundity estimate for the York River from Hyle et al. [14], and new indeterminate fecundity estimate for the Connecticut River from the current study (new determine fecundity estimate not shown).

The indeterminate-method estimates proved redundant to the determinate-method estimates for the Connecticut River population of American Shad (Fig 9). Inter-decadal comparisons suggest some level of stability in the fecundity-size relationships in the Connecticut River, in the range of 250–300 thousand eggs produced by a young (virgin, approximately age-5) female. Prior to the initiation of fish passage at the Hadley Power Station in the 1950’s, the entire population of Connecticut River American shad spawned in the limited area below the dams. During the 1960’s, population estimates varied from 367,000 to 1,470,000 fish (mean ± sd = 630,000 ± 365,000) with limited upstream passage [32]. Since that time, passage at Hadley Power Station has increased, with hundreds of thousands of American shad moving upriver each year. Thus, population size varies from year to year but appears to be at the same order of magnitude since the 1960s. Nonetheless, improvements to fish passage mean that the total spawning area has increased, producing a decreased density of spawners per river kilometer. American shad fecundity data at present are limited, for all populations, but future researchers should be aware of the potential for within population, year-to-year PAF variability driven by abiotic or biotic factors against the backdrop of a latitudinal trend among populations. Annual fecundity in some species varies from year to year [33, 34], and as much as 10 fold over longer periods [35], for some data-rich examples.

American shad spawns within its native range from Maritime Canada to Florida, USA (50–30°N), and is defined by dramatic intra-specific variation in life history traits [5, 28]. One of these patterns is decreasing annual fecundity with increasing latitude, but the average number of lifetime spawning migrations also increases with latitude, so that lifetime fecundity is predicted to be stationary across latitudes. The current study working with the Connecticut River population found that annual fecundity was not different by method, which suggested that the determinate fecundity method can be used routinely and that historic estimates are likely accurate–at least for this population. This was not the conclusion reported for the York River, Virginia, population [14], where evidence of asynchronous oocyte development was observed and use of an indeterminate fecundity method produced much higher estimates–twice as high as previous reports. A working hypothesis is that the mode of yolked oocyte development is group synchronous in the north but asynchronous in the south. If so, the latitudinal gradient in American shad annual fecundities may be greater than previously estimated (Fig 9). Additional comparisons between methods, along a latitudinal gradient is necessary to accept this postulation, but if true, it would preserve the argument for adaptive significance of life history variation and natal homing in this species, while leading to new understandings into sources of fecundity variation between populations, years, and individuals.

## Supporting Information

S1 Material Ferreri R, McBride RS, Towle EK, Basilone B. **Oocyte distributions from American shad in two southern New England (USA) rivers: the Connecticut and Merrimack rivers.** Supplemental materials to: McBride et al., “Yolked oocyte dynamics support agreement between determinate- and indeterminate-method estimates of annual fecundity for a northeastern United States population of American shad”.
(PDF)
