# Supplementary material for: Yolked Oocyte Dynamics Support Agreement between Determinate- and Indeterminate-Method Estimates of Annual Fecundity for a Northeastern United States Population of American Shad
Source: PLoS One. 2016 Oct 7;11(10):e0164203. doi: 10.1371/journal.pone.0164203 (PMC5055366; doi:10.1371/journal.pone.0164203)
Supplement: S1 Material — Oocyte distributions from American shad in two southern New England (USA) rivers: the Connecticut and Merrimack rivers. Supplemental materials to: McBride et al., “Yolked oocyte dynamics support agreement between determinate- and indeterminate-method estimates of annual fecundity for a northeastern United States population of American shad”. (PDF) [file pone.0164203.s001.pdf]

Ferreri, Rosalia, Richard S. McBride, Emilee K. Towle, and Gualtiero Basilone. 2015. Oocyte distributions from American shad in two southern New England (USA) rivers: the Connecticut and Merrimack rivers.

Supplemental materials to McBride et al. (2016). Yolked oocyte dynamics support agreement between determinate- and indeterminate-method estimates of annual fecundity for a northeastern United States population of American shad. PLoS One.

A preliminary set of collections occurred in 2014 to evaluate between-river and within-river variation of oocyte development. American shad were sampled at the first (i.e., most downstream) hydroelectric power station encountered on the main stem of two rivers: the Connecticut River, which flows into Long Island Sound of the U.S. middle Atlantic seaboard, and the Merrimack River, which flows into the southern Gulf of Maine. Three dates were sampled in each river; each date was designed to capture fish at roughly the beginning, middle, and end of the spawning run.

Relative to the entire latitudinal range of American shad, from the Canadian maritime to Florida, USA, both rivers are neighbors. Each river passes through Massachusetts. Nonetheless, the linear distances between each river mouth is about 240 km, and fish migrating to the Connecticut River presumably travel a greater distance from the offshore feeding grounds. Therefore, some variation in oocyte development may be observed between fish spawning in these two rivers.

We evaluate the synchrony of oocyte development by examining the gonad histology and by measuring the diameters of whole oocytes of fish selected from each sampling date in each river.

Methods

Field methods. Female American shad were collected at fish passage operations in two rivers: at the Hadley Falls Power Station, in the City of Holyoke, Massachusetts, on the Connecticut River (42.2°N, 72.6°W), and the Lawrence Hydro Plant, on Bodwell's Falls, in the City of Lawrence, Massachusetts, on the Merrimack River (42.7°N, 71.2°W). All fish were moving upstream when collected from the lift and trap operations at each power station.

Fish were collected from morning to early afternoon, generally from 1000 to 1400, on three dates that represented early, middle, and late portions of the 2014 spawning run. A total of 30 females were targeted on each day; realized sample size ranged from 9 to 30 females in the time allowed each day (Fig A).

Laboratory methods. Fish were processed within 24 hours to ensure the quality of the reproductive tissue. Size, reproductive traits, and age were measured as stated in the main text to this supplemental material (McBride et al., 2016).

Observations were made from histological preparations and from estimates of whole oocyte diameters (Figs B, C; see also McBride et al., 2016); both methods used ovarian tissue fixed in buffered formalin. All females collected in 2014 were captured in upstream fish passage systems, where we collected a variety of maturity classes:

developing (Class A), hydrated (Class B), and partially spent (Class D) (See main text describing maturity classes). Two replicate fish, one younger and one older, were selected from developing (Class A) fish on each sampling date at both rivers, for a total of 12 fish. An additional six fish were selected to observe oocyte sizes associated with the process of oocyte maturation, as observed in ripe females (Class B), as well as ovarian regression, as observed in partially-spent females (Class D).

Results

Adult females were collected on all sampling dates spanning 6 weeks in each river. They ranged from 365 to 520 mm fork length (L_F) (746 – 2020 g total body mass (M_T) in the Connecticut River, and from 418 to 527 mm L_F (968 – 1987 g M_T) in the Merrimack River (Fig A).

At the whole gonad level, all females were characterized as developing in each river on the first sampling date (12.0 – 26.2 gonad-somatic index (I_G) Conn. River; 12.3 – 17.3 I_G Merr. River). On the second sampling date, some females were hydrated, and the rest were developing (10.7 – 22.0 I_G Conn. River; 8.1 – 21.2 I_G Merr. River). On the third sampling date, three maturity classes were observed: developing, hydrated, and partially spent (10.7 – 20.8 I_G Conn. River; 5.2 – 20.1 I_G Merr. River).

At the whole oocyte level, a total of four oocyte phases were observed: 1) small (< 0.3 mm), clear oocytes (presumed to be perinucleolar), 2) medium (0.3 – 0.5 mm), grayish oocytes (presumed to be cortical alveolar stage), 3) a broad range (0.5 – 1.5 mm) of opaque oocytes (presumed to be vitellogenic stage), and the largest range (0.15 – 2.0 mm) of translucent or transparent oocytes (presumed to be mature stage, including hydrated

oocytes). These presumptions about which histological stage matched which whole oocyte phase were confirmed by direct comparison of stage and phase for individual fish examined here, as well as in subsequent collections in 2015 (McBride et al., 2016).

At Hadley Falls, Connecticut River, developing females sampled in May or June were ready or had begun to spawn. Two principal size groups of whole oocytes were evident, associated with either a tightly clustered cohort of perinucleolar (PE) oocytes or a broad range of vitellogenic (V1, V2) and early mature (NM1) oocytes (Fig D). The size gap between these two modes was not as obvious as might be expected for a fish with group-synchronous yolked oocyte development. When evident, the gap in sizes was in the 0.3 – 0.5 mm range, which was associated with the cortical alveolar (C1, C2) stage of oocyte development (yellow colored histogram, with black angled pattern overlaid, in Fig D). When not evident, there was a reduced number of germ cells in this size range (i.e., an antimode), suggesting that de novo vitellogenesis had stopped.

Similar results were obtained also for Bodwell’s Falls, on the Merrimack River. In most fish, a gap in sizes was evident in the 0.3 – 0.5 mm range (Fig E).

The size gap between previtellogenic and vitellogenic oocytes was not large but fairly unambiguous in the ripe fish collected in both rivers (Fig F). An advanced cohort of oocytes in late maturation (NM3) or even hydration was also apparent, demonstrating the group-synchronous nature of oocyte maturation in American shad. Histological analysis results confirmed that only one female had fully hydrated oocytes.

In the two partially spent fish (ASAP 154, 158), oocytes in early maturation (NM1) indicated that spawning was continuing. In at least one female (ASAP 154) a bimodal

distribution of vitellogenic oocytes and a cohort of recrudescing cortical alveolar staged oocytes were recognizable.

Discussion

Yolked oocyte development in Connecticut River and Merrimack River American shad appears to be group synchronous. A size gap, at about 0.3 – 0.4 mm, was evident in the oocyte distributions of the majority of upstream migrating females. This size gap corresponded to previtellogenic oocytes with cortical alveoli (C1, C2). When a specific size gap was not evident, the number of C1 and C2 oocytes were fewer (i.e., an antimode) than the smaller (PE) and the larger (V1, V2) oocytes. The presence or size of the gap did not appear associated with respect to female size or age. McBride et al. (2016) point out an association of the presence or size of the gap with respect to maturity class or date: developing females (Class A) early in the spawning run have few or no transitional growth phase (C1, C2) oocytes, but later, spent and resting females (Class E, F) have these oocyte stages as a result of ovarian recrudescence.

The actual size gap was not as large as might be expected. In the Atlantic herring (*Clupea harengus*) the reported size gap varies from only slightly wider than observed here (0.2 – 0.3 to 0.6 – 0.7 mm; Hickling and Rutenberg, 1936; Hay 1985) to a much larger gap (0.2 – 0.3 to 1.2 – 1.4 mm; Kurita et al. 2003). A size gap may have been more obvious in the Merrimack River than in the Connecticut River fish, but this was not thoroughly investigated. More detailed characterization of oocyte growth dynamics among cells < 500 μm would be informative but would require specialized techniques (e.g., Kurita and

Kjesbu, 2009; Schismenou et al., 2012; Saber et al., 2016). Also, applying this approach to include fish from a broader range of river systems should help address if synchrony of oocyte development varies systematically along a latitudinal gradient, as observed for other life history traits by this species (McBride et al., 2016).

Oocyte development in Connecticut River and Merrimack River American shad appears to be group synchronous with respect to oocyte maturation, as well. Nucleus migration appears to start in oocytes in the 1.2 – 1.5 mm range, and a batch advances as a group with whole, hydrated oocytes reaching 1.5 – 2.0 mm in diameter. The numbers of smaller vitellogenic oocytes were diminished in partially spent fish, indicating that the stock of vitellogenic oocytes was being depleted with each subsequent spawning event; however, sampling in 2014 did not capture downstream migrating, spent or resting fish to clearly examine this phenomenon (but see McBride et al., 2016).

Group synchronous development with respect to maturation and ovulation confirms that American shad are batch spawners. Lehman (1953) clarified the differences in batch and annual fecundity. Lehman (1953) appears to be the first to have appropriately estimated annual fecundity – in the 100s of thousands of eggs – whereas earlier estimates were of batch fecundity, originating primarily from hatchery studies – in the 10s of thousands of eggs. In 2014, evidence of spawning, in both the form of hydrated and partially spent females, was observed at the first hydro-electric power station on both rivers as early as mid- to late May.

American shad has a fairly large egg, compared to marine fishes, but this is not unusual relative to its congeners or other freshwater spawners (Teletchea et al. 2009).

In summary, American shad oocyte development in these two rivers appeared to be group synchronous, at the transitions of yolk development and maturation. This conclusion is based on a preliminary sampling effort in 2014. Sampling in 2015 was designed to sample fish across a greater spatial range within the river and therefore represent a greater range of maturity at the beginning and end of the spawning run.

References

Hay, D.E. (1985) Reproductive biology of Pacific herring (*Clupea harengus pallasi*). *Canadian Journal of Fisheries and Aquatic Sciences* 42, 111-126.

Hickling, C.F., Rutenberg, E. (1936) The ovary as an indicator of the spawning period in fishes. *Journal of the Marine Biological Association of the United Kingdom* 21, 311-317.

Kurita, Y., Meier, S., Kjesbu, O. (2003) Oocyte growth and fecundity regulation by atresia of Atlantic herring (*Clupea harengus*) in relation to body condition throughout the maturation cycle. *Journal of Sea Research* 49, 203-219.

Kurita Y, Kjesbu OS. Fecundity estimation by oocyte packing density formulae in determinate and indeterminate spawners: theoretical considerations and applications. J Sea Res. 2009; 61: 188–196. doi:10.1016/j.seares.2008.10.010

Lehman, B.A. (1953) Fecundity of Hudson River shad. *United States Fish and Wildlife Service, Research Reports* 33, 1-8.

McBride, R.S., R. Ferreri, E.K. Towle, J. M. Boucher, G. Basilone. (2016). Yolked oocyte dynamics support agreement between determinate- and indeterminate-method estimates of annual fecundity for a northeastern United States population of American shad. PLoS One.

Saber S, Macías D, de Urbina JO, Kjesbu OS. Contrasting batch fecundity estimates of albacore (*Thunnus alalunga*), an indeterminate spawner, by different laboratory techniques. Fish Res. 2016; 176: 76-85. doi: 10.1016/j.fishres.2015.12.013

Schismenou E, Somarakis S, Thorsen A, Kjesbu OS. Dynamics of de novo vitellogenesis in fish with indeterminate fecundity: an application of oocyte packing density theory to European anchovy, *Engraulis encrasicolus*. *Marine Biology* 2012; 159: 757–768. doi:10.1007/s00227-011-1852-y

Teletchea, F., Fostier, A., Kamler, E., Gardeur, J.N., Le Bail, P.Y., Jalabert, B., Fontaine, P. (2009) Comparative analysis of reproductive traits in 65 freshwater fish species: application to the domestication of new fish species. *Reviews in Fish Biology and Fisheries* 19, 403-430.

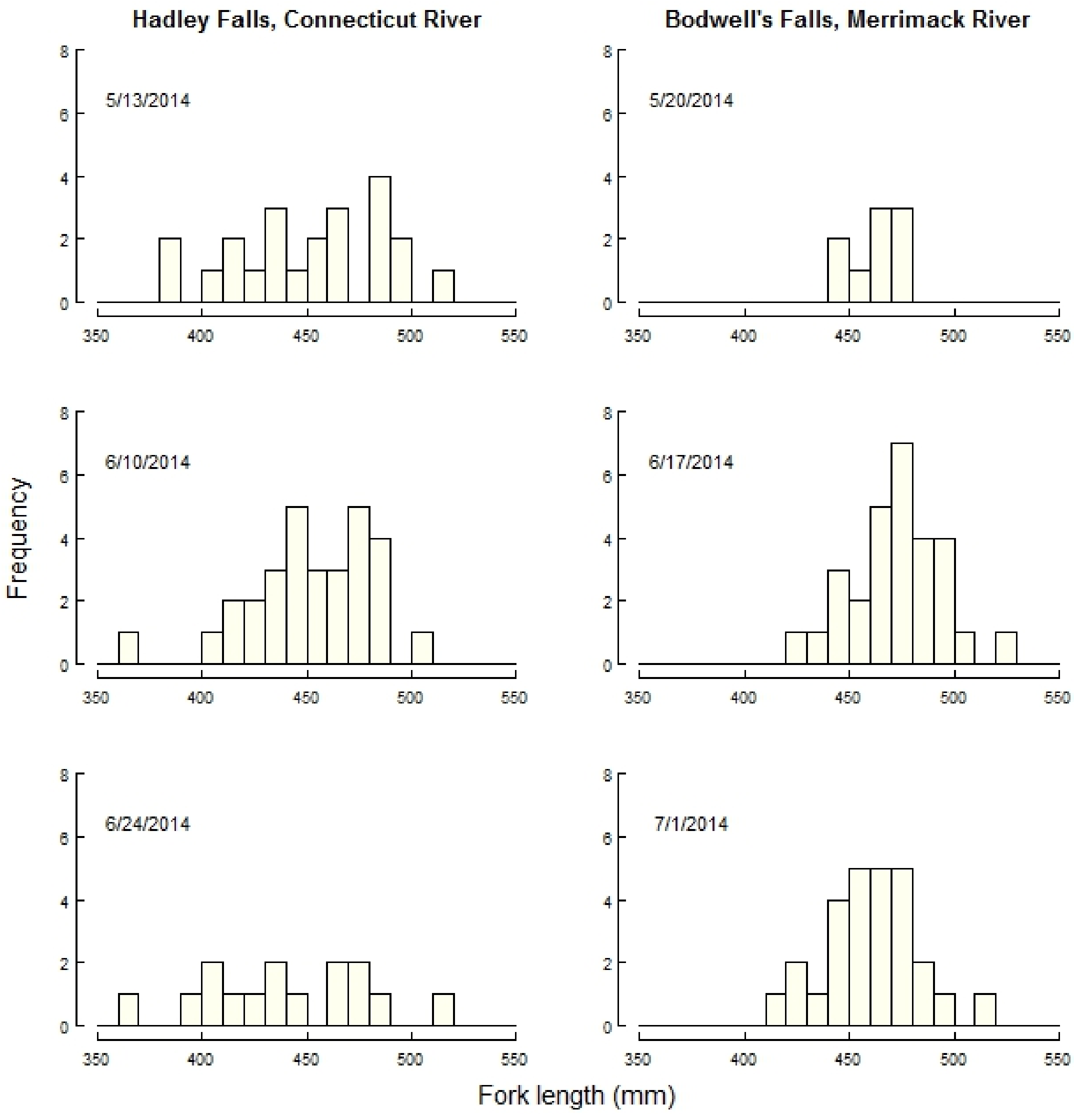

Fig A. Size distribution of female American shad at all six sampling events: Connecticut River (left) and Merrimack River (right).

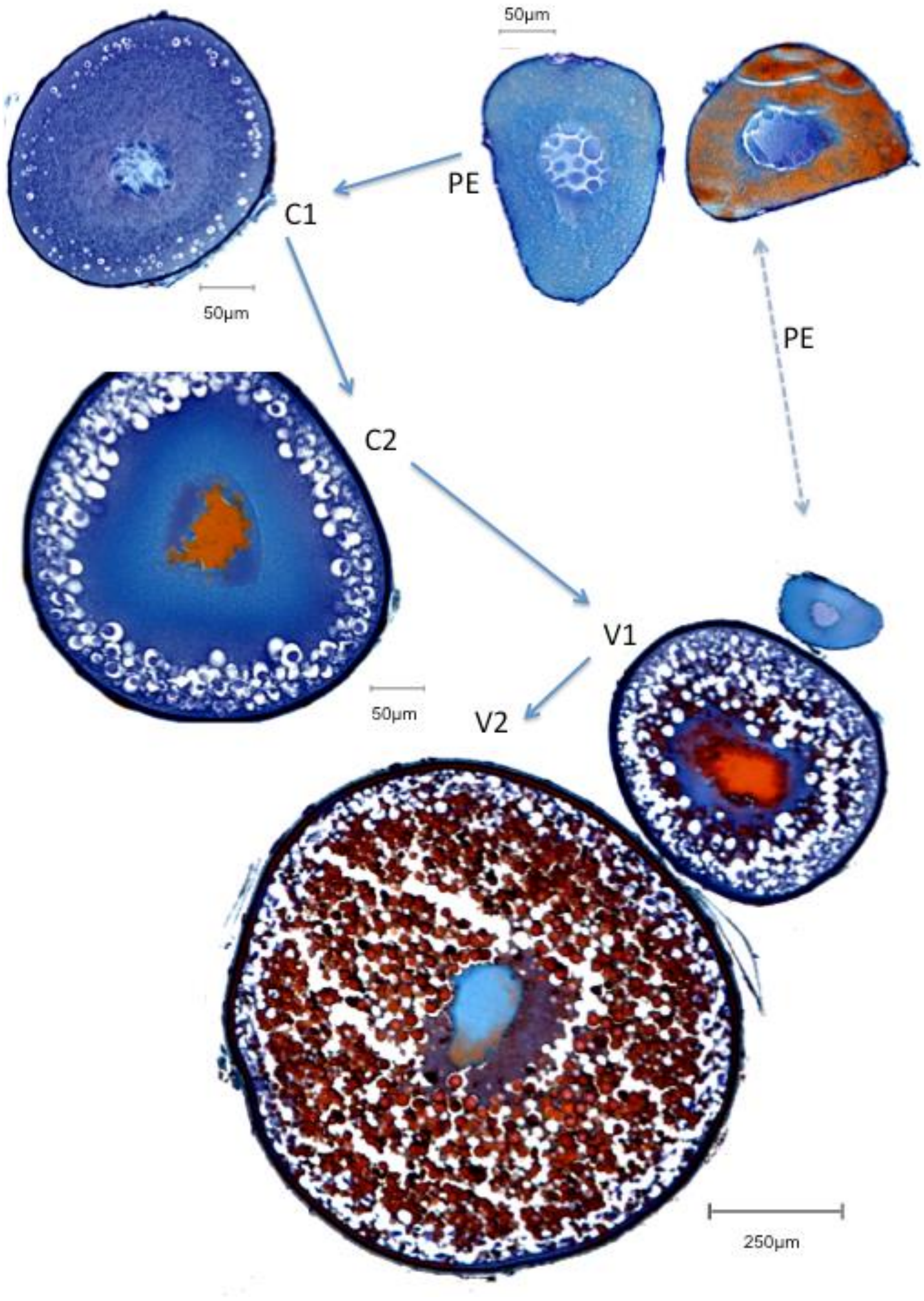

Fig B. Histology images of the oocyte transition from primary growth (perinucleolar phase: PE), to transitional growth (cortical alveoli: C1 and C2), to secondary growth (vitellogenesis: V1 and V2). The scale of vitellogenic stages is different from the others pictures (PE, C1 and C2).

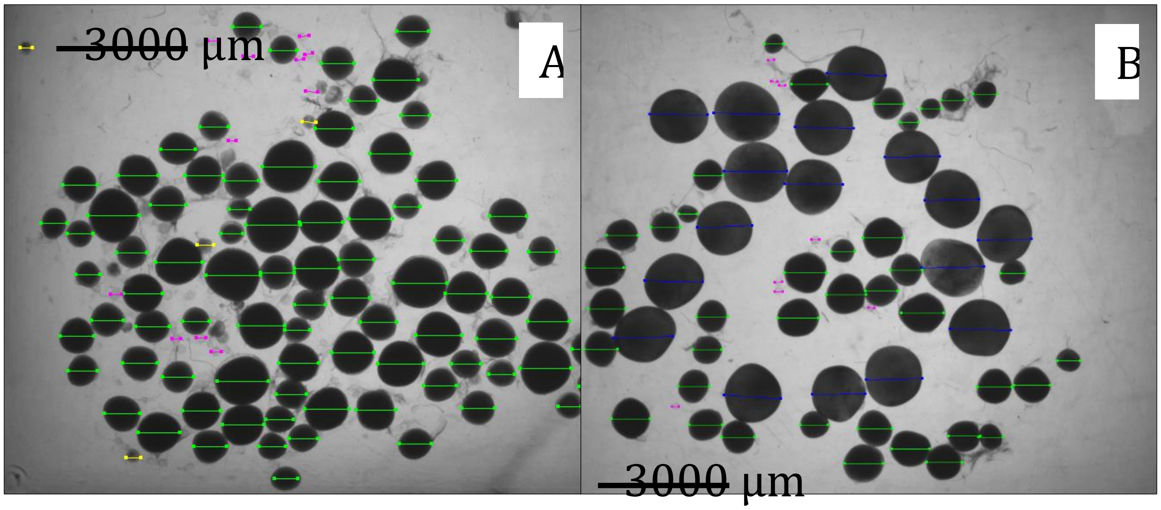

Fig C. Images of whole oocytes from one fish with vitellogenic oocytes (A) and one fish with hydrated oocytes (B) as the most advanced stage. (A) Three different maturity stages of oocytes appear: earliest oocyte with perinucleolar (PE – transparent cells, with measuring bar in pink); cortical alveoli (C1 and C2 – translucent, gray cells, with measuring bar in yellow); vitellogenic oocytes (V1 and V2 – opaque, black cells, with measuring bar in green). (B) This also shows hydrated eggs (H – translucent, gray cells, with measuring bar in blue).

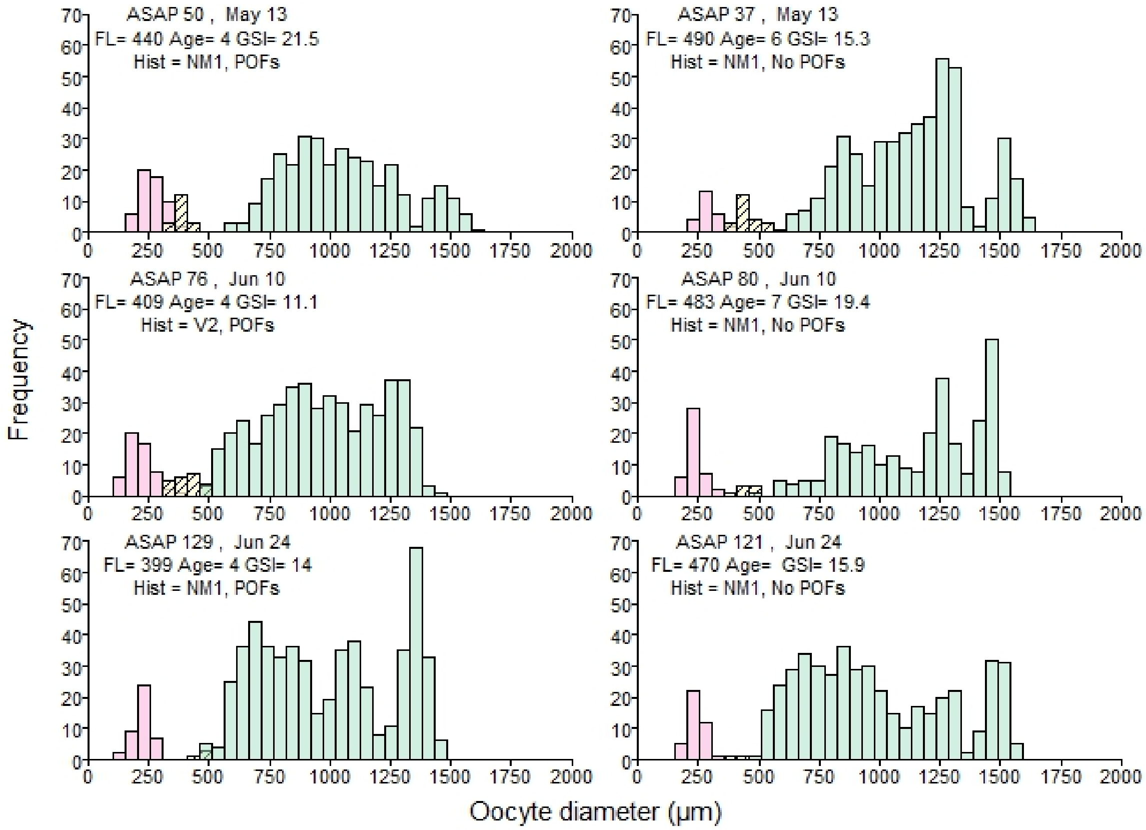

Fig D. Histograms of oocyte diameters of female American shad collected from the Connecticut River in 2014. All six fish were developing (macroscopic maturity class A), displaying a smaller, younger fish (left) and a larger, older fish (right) collected on three different dates. Labels include: a unique ASAP number, sampling date (2014), fish length (fork length, mm), fish age (years, otolith method), gonad-somatic index (GSI), and histology details (i.e., most advanced oocyte [see text and Fig. 2 for definitions] and whether post-ovulatory follicles were present or absent). Colors are transparent (not stacked), corresponding to small, translucent oocytes (red), medium, translucent oocytes (yellow), and larger, opaque oocytes (green). Angled hatching is overlaid on the yellow bars to distinguish them more. There is no age for ASAP 121.

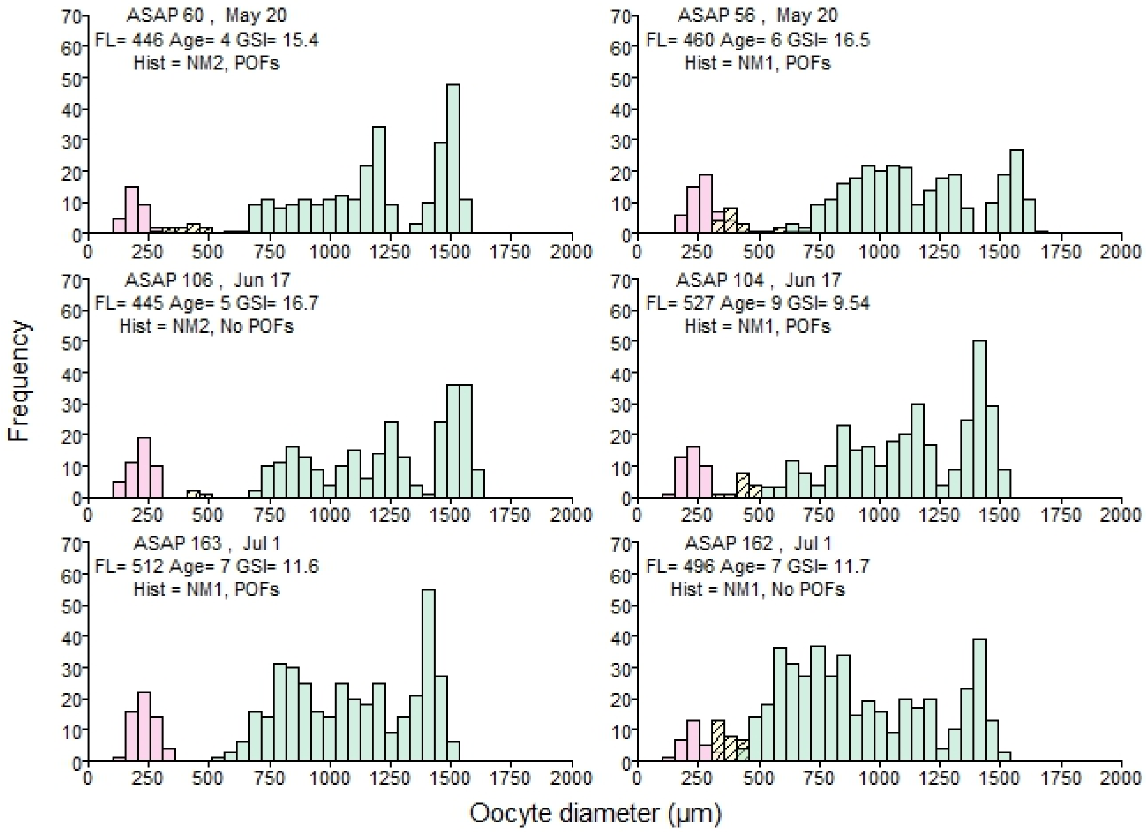

Fig E. Histograms of oocyte diameters of female American shad collected from the Merrimack River in 2014. All six fish were developing (maturity class A), displaying a smaller, younger fish (left) and a larger, older fish (right) collected on three different dates. Labels and colors are the same as in Fig D.

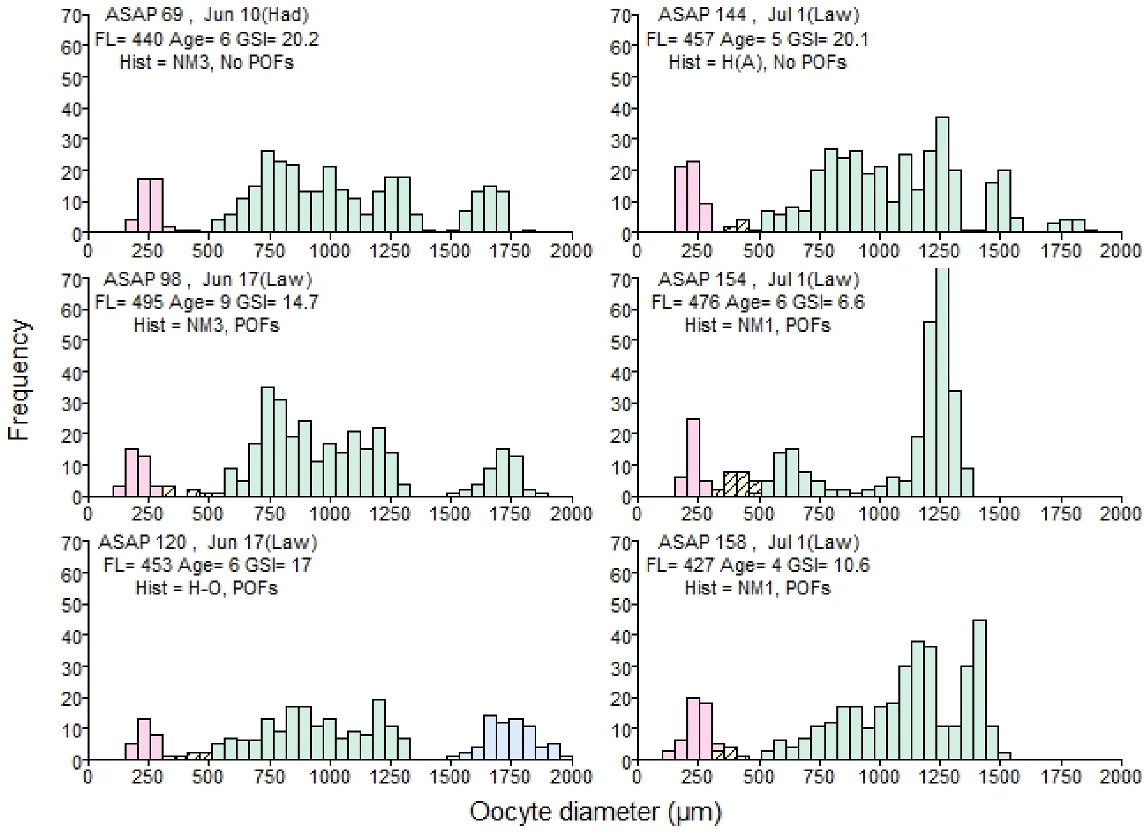

Fig F. Histograms of oocyte diameters of female American shad collected in 2014 from Hadley Falls Power Station (Had) on the Connecticut River or the Lawrence Hydro Plant (Law) on the Merrimack River. Fish are selected to show the advanced mode of mature oocytes (1.5 – 2.0 mm) in three ripe fish (maturity class B; left). On the right, ASAP 144 is unusual, appearing to spawn for the first time but some hydrated oocytes are atretic; the other two fish (ASAP 154, 158) are partially spent (maturity class D), so spawning has started and is continuing in these females. Labels and colors are as in Fig D, with the addition of blue for the hydrated (H) or ovulated (O) oocytes in ASAP 120.
